# Structure of Langmuir Monolayers of Perfluorinated Fatty Acids: Evidence of a New 2D Smectic C Phase

**DOI:** 10.3390/molecules24193590

**Published:** 2019-10-05

**Authors:** Philippe Fontaine, Eduardo J. M. Filipe, Marie-Claude Fauré, Tomas Rego, Stephanie Taßler, Ana Carolina Alves, Gonçalo M. C. Silva, Pedro Morgado, Michel Goldmann

**Affiliations:** 1 Synchrotron SOLEIL, L’Orme des Merisiers, Saint Aubin, BP48, 91192 Gif sur Yvette CEDEX, France; philippe.fontaine@synchrotron-soleil.fr (P.F.); stephanie.tassler@web.de (S.T.); 2 Centro de Química Estrutural, Instituto Superior Técnico, Universidade de Lisboa, 1049-001 Lisboa, Portugal; efilipe@ist.utl.pt (E.J.M.F.); carolina.o.alves@gmail.com (A.C.A.); goncalo.silva20@gmail.com (G.M.C.S.); pm.elessar@gmail.com (P.M.); 3 Institut des NanoSciences de Paris, UMR 7588 CNRS, Sorbonne Université, 4 place Jussieu, 75252 Paris CEDEX, France; Marie-Claude.Faure@insp.jussieu.fr (M.-C.F.); tomas.rego@insp.jussieu.fr (T.R.); 4 Faculté des Sciences Fondamentales et Biomédicales, Université de Paris, 45 rue des Saints-Pères 75006 Paris CEDEX, France

**Keywords:** perfluorinated amphiphiles molecules, Langmuir monolayer, 2D phase diagram

## Abstract

Due to the characteristic chain rigidity and weak intermolecular interactions of perfluorinated substances, the phase diagram of Langmuir monolayer formed by perfluorinated molecules has been interpreted so far as displaying only two phases, a 2D gas (G) and a liquid condensed (LC). However, in this work, we presented Grazing Incidence X-ray Diffraction measurements, which exhibit two diffraction peaks on the transition plateau: One is the signature of the hexagonal structure of the LC phase, the second one is associated to the low-density fluid phase and is thus more ordered than expected for a 2D gas or a typical fluid phase. Atomistic molecular dynamics simulations, performed on the transition plateau, revealed the existence of clusters in which domains of vertical molecules organized in a hexagonal lattice coexist with domains of parallel lines formed by tilted molecules, a new structure that could be described as a “2D smectic C” phase. Moreover, the diffraction spectrum calculated from the simulation trajectories compared favorably with the experimental spectra, fully validating the simulations and the proposed interpretation. The results were also in agreement with the thermodynamic analysis of the fluid phase and X-ray Reflectivity experiments performed before and after the transition between these two phases.

## 1. Introduction

Perfluorinated materials display specific properties, which confer their potential interest for applications in many domains. Perfluorinated surfactants, for example, are widely used as solvents, reaction promoters, refrigerants, pharmaceutical reactants, and other [1]. When spread at the surface of a solid substrate, they modify the surface physical and chemical properties [2,3]. Given their biocompatibility and non-toxicity, perfluorinated substances are also considered for biological applications, such as oxygen transport (blood substitute) [4], drug delivery [5], and other biomedical research [6,7,8].

Structurally, perfluorinated chains display a wider cross-section (0.283 nm^2^ compared to 0.185 nm^2^ for hydrogenated chains) [9] and consequently higher densities and molar volumes than hydrogenated chains with the same number of carbon atoms. Another important difference between perfluorinated and hydrogenated chains is conformational. For perfluorinated chains, the dihedral angle at minimum energy is not exactly 180 degrees, as it is for hydrogenated. Consequently, perfluorinated chains adopt a characteristic helical conformation, unlike hydrogenated chains that tend to be in their all-trans planar form [10]. Additionally, the energy barrier to internal rotation of perfluorinated chains is much higher than that of hydrogenated that induce a rigid character, in contrast with the flexible character of hydrogenated chains [11]. These features have well-known consequences. Fluorinated alcohols, for example, display lower melting enthalpies (ΔH_fus_) and melting entropies (ΔS_fus_) than their hydrogenated counterparts, and higher melting temperatures [12,13]. They are also known to be more volatile. Perfluorinated substances thus display smaller liquid ranges than their hydrogenated analogs. In other words, the transition from the liquid to the solid phase is easier.

The usefulness of Langmuir monolayers formed by insoluble amphiphilic molecules deposited at the air-water interface has been widely established as model systems in the field of bi-dimensional fundamental physics [14,15]. These films are successfully used to study phase’s transitions and structures in reduced dimensionalities, thanks to various experimental techniques, such as thermodynamic (surface pressure measurements), optical microscopy, X-ray Reflectivity (XRR), and Grazing Incidence X-ray Diffraction (GIXD). However, although the main features of the phase diagram of the simplest amphiphiles, such as fatty acids or alcohols, are well understood [15], the monolayer behavior of perfluorinated amphiphiles are not completely characterized. We have previously demonstrated [16,17] by GIXD that the critical temperature of the observed and so-called 2D gas (G)-liquid condensed (LC) transition is lower for perfluorinated fatty acids than for the more common hydrogenated analogs. This is in agreement with the higher-pressure value observed for the plateau of this Fluid-LC transition and has been justified by the rigidity of the chain associated with the weaker intermolecular interactions between fluorinated chains [18].

The successive phase transition observed in adsorbed surfactant monolayers, e.g., fatty acids at the surface of the water, is a classic problem in physical chemistry. Many theoretical approaches have been developed in an effort to account for the ordering transitions (position and/or orientation) in these systems and predict the first or second-order character and the relevant order parameter to describe these phase transitions. Computer simulations provide a powerful alternative way to answer such questions, especially given the fast growth of computing power in recent years.

In this work, we reported a combined macroscopic and microscopic study focused on the Langmuir monolayer of perfluorododecanoic acid (PFDA, C_11_F_23_COOH). In addition to the usual π-A isotherms and XRR measurements, we used the higher resolution of GIXD available on the SIRIUS beam-line at the SOLEIL Synchrotron [19] to determine more accurately the monolayer structure and phase transition on the “Fluid-LC” transition plateau. We have been able to describe quantitatively the fluid phase, for the first time, which displays a limited organization as Liquid Crystal phases that can be considered as similar to a 2D smectic C phase. This description is supported by new atomistic Molecular Dynamics (MD) simulations, which provide a molecular-level interpretation of the experimental results and a visualization of the structure of the monolayer. The simulations are validated by the diffraction pattern calculated from the simulation trajectories, which reproduce the experimental X-ray spectrum.

## 2. Results

### 2.1. Thermodynamic Study

**Figure 1:**
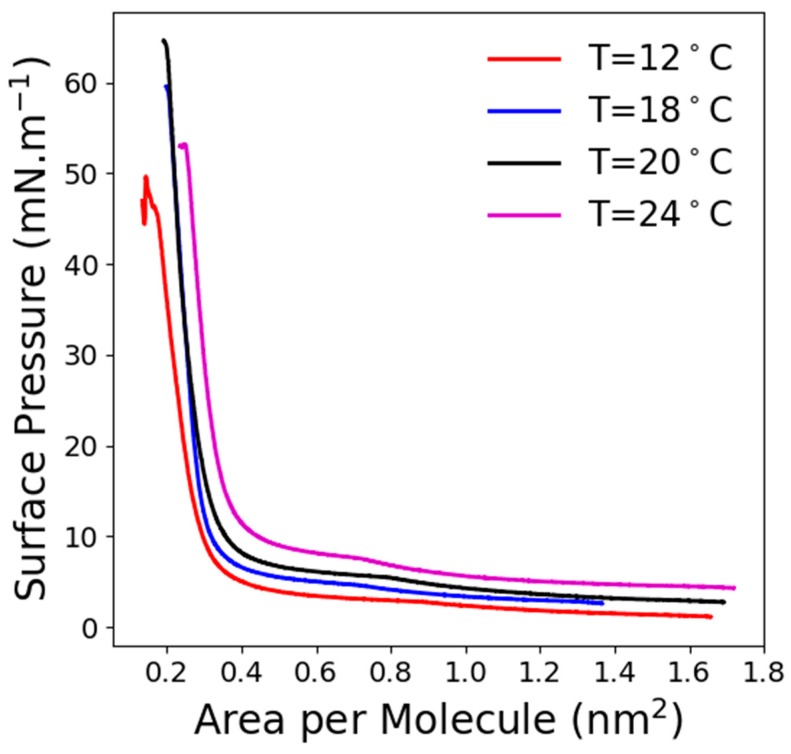
Surface pressure-area per molecule (π-A) isotherms of C_11_F_23_CO_2_H at various temperatures.

The surface pressure-area per molecule (π-A) isotherms of C_11_F_23_CO_2_H monolayer for different temperatures is presented in Figure 1. These isotherms presented, at low surface density, a weak pressure increase up to about 0.8 nm^2^ per molecule, depending on the temperature, followed by a weak slopping surface pressure plateau (varying from 3 to 9 mN/m with a temperature increase from 12 to 24 °C) and finally a steep pressure increase. Such (π-A) isotherm reflected a first-order phase transition between a fluid phase at a large area per molecule and a condensed phase designated as liquid condensed (LC).

The compressibilities of these two phases at different temperatures, calculated using the usual relation $\chi =-\frac{1}{A}\left(\frac{\partial A}{\partial \pi }\right)$, are presented in Table 1. The compressibility of the fluid phase was over 130 m/N and that one of the LC phase was under 10 m/N, differing by more than one order of magnitude.

### 2.2. X-ray Reflectivity

**Figure 2:**
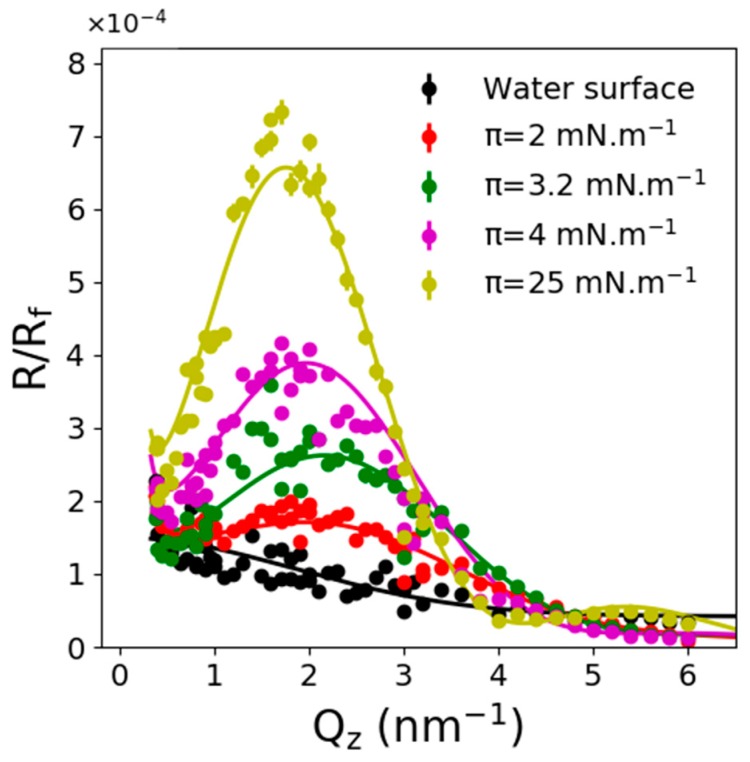
Fresnel normalized X-ray reflectivity spectra of C_11_F_23_CO_2_H monolayers at 20 °C and several pressures below and above the Fluid-LC (liquid condensed) phase transition plateau.

**Figure 3:**
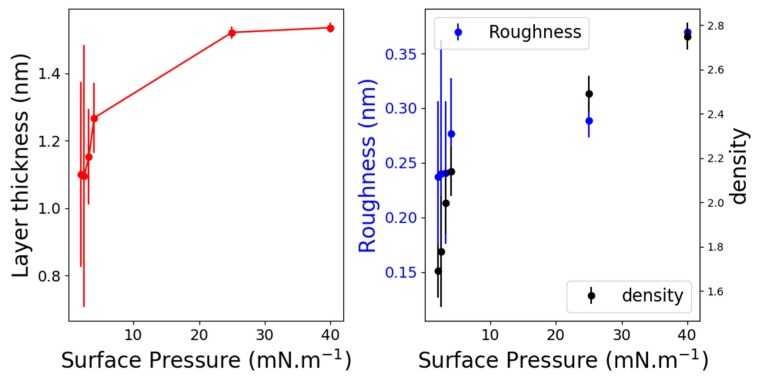
Thickness (left) and roughness and density (right) results from a single layer adjustment of the X-ray reflectivity.

Figure 2 presents the reflectivity spectra (normalized by the Fresnel one) of C_11_F_23_CO_2_H monolayers at 20 °C obtained at several pressures above and below the Fluid-LC phase transition plateau. The monolayer can thus be considered as being in a monophasic state in all measurements. Note that the high scattering power of perfluorinated chains allows detecting reflectivity signals for area per molecules up to 10 nm^2^/molecule. No minimum was observed below 0.4 nm^−1^ on these spectra, indicating a low thickness of the contrasting layer. This confirmed the monolayer state of the film. The calculated thickness obtained modeling the film as a single layer for various surface pressure is presented in Figure 3. In the LC phase (π > 20 mN/m), the thickness remained constant around 1.5 nm, which was in very good agreement with a vertical orientation of the chains. Indeed, the length of the extended molecule was estimated at 1.7 nm that could be divided as follows; 1.4 nm for the fluorinated chain and 0.29 nm for the acid head-group. The resulting density varied from 2.5 to 2.75 as the surface pressure increased from 25 mN/m to 40 mN/m. This density variation, associated with the constant thickness of the film, was in very good agreement with the surface density evolution (0.29 to 0.255 nm^2^/molecule). These values seemed also reasonable considering the 2.16 density of the PTFE polymer.

On the contrary, one observed in the fluid phase that the thickness of the monolayer increased from 1.1 to 1.3 nm, corresponding to an average tilt angle for the chains of 42° to 30°, respectively (using the 1.5 nm thickness as the reference for the vertical molecule). The film density increased from 1.5 up to 2.4 when the surface pressure varied from 2 to 4 mN/m (corresponding to the molecular area of 1.7 and 1.1 nm^2^/molecule, respectively). The surface roughness increased from 0.24 nm for the fluid phase to 0.35 nm for the LC one. This could be justified by the fact that the surface tension is lower in the LC phase, leading to weaker damping of the capillary wave height fluctuations. These values which range within 0.3 nm are observed on pure water [20,21].

### 2.3. Grazing Incidence X-ray Diffraction

**Figure 4:**
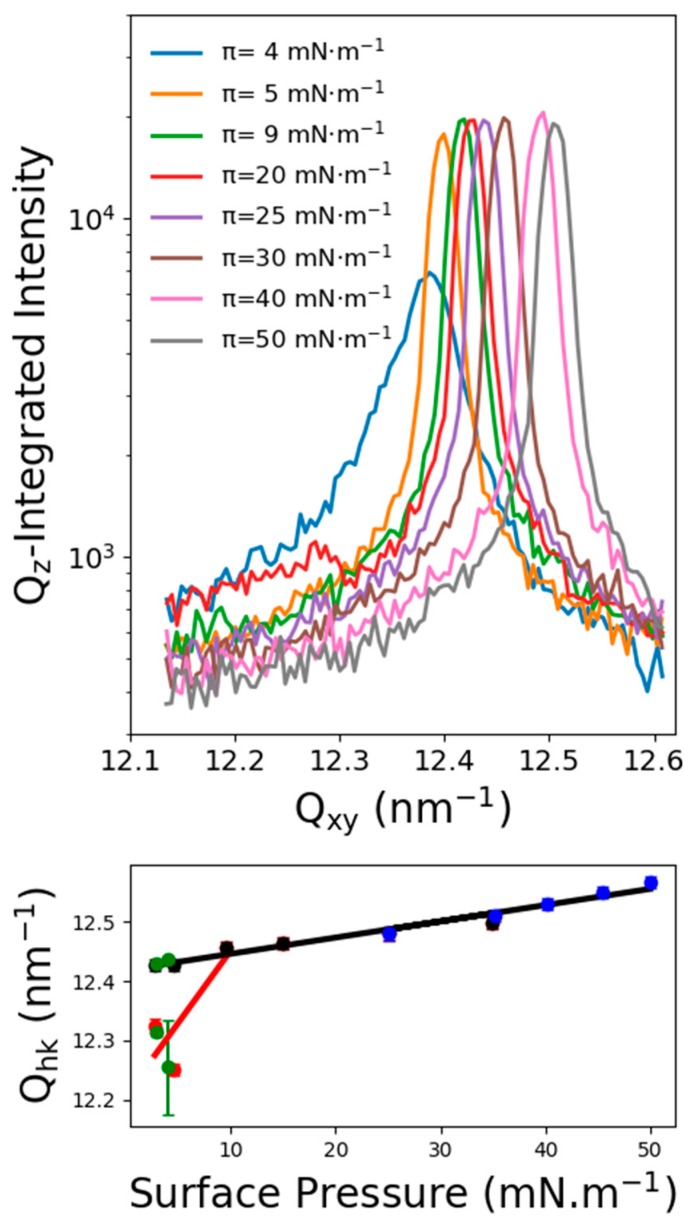
Top: Qz integrated diffraction intensity of C11F23CO2H at 18 °C at different surface pressures. Bottom: (11) and (02) diffraction peak’s positions, resulting from one or two peaks adjustment with respect to the surface pressure.

Figure 4-top depicts the GIXD spectra of C_11_F_23_CO_2_H at 18 °C and for different surface pressures. The spectra were different whether they were recorded on the Fluid-LC transition plateau or in the LC phase; for example, spectrum on the transition plateau.

**Figure 5:**
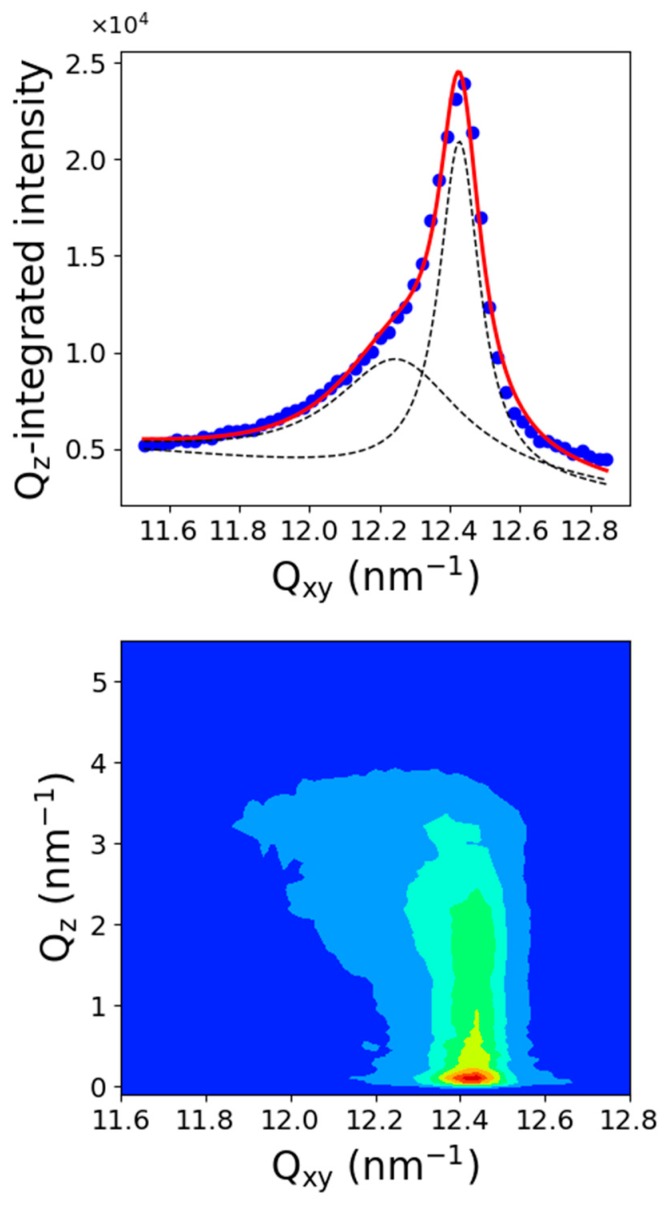
Diffraction spectra of C_11_F_23_CO_2_H at 18 °C on the Fluid-LC transition plateau.

Figure 5 presents the Q_z_-integrated spectrum and the corresponding Q_xy_, Q_z_ diffraction map. The spectrum was dissymmetric and must be fitted by using two Lorentzian functions, one centered at Q_xy_ = 12.25 ± 0.03 nm^−1^ and another one at Q_xy_ = 12.42 ± 0.01 nm^−1^. The Q_xy_-Q_z_ intensity map shows that the Q_z_ maximum of the lower Q_xy_ diffraction peak, although not well defined, was located out-of-the plane at about Q_z_ = 2.5 ± 0.1 nm^−1^. The maximum intensity of the peak at higher Q_xy_ was located in the plane at Q_z_ = 0 nm^−1^. Figure 4-bottom presents the peaks maximum evolution (fitted with one or two Lorentzian functions) with respect to the surface pressure. Two peaks were observed up to 9 mN/m, which corresponds to the end of the Fluid-LC transition plateau. A first possible interpretation would be to associate such a two diffraction peaks pattern to a single structure. In this case, the lower Q_xy_ peak was indexed (11) and the higher Q_xy_ peak (02), using a 2D rectangular cell containing two molecules. The corresponding lattice parameters were a = 0.595 nm and b = 1.011 nm, leading to a unit cell area of 0.601 nm^2^ and an area per molecule of 0.3 nm^2^. However, the weak resolution of the rod profile of the (11) peak with respect to that of the (02) peak is questionable. Also, the intensities ratio between the two peaks does not seem to remain constant. Then, another possibility is that this spectrum reflects the coexistence of two structured phases, each one leading to a diffraction peak. In this case, the peak at Q_xy_ = 12.42 nm^−1^ corresponded to the single peak obtained at high pressure in the single LC phase. Although this interpretation appears as more satisfying, one must determine the origin of the other diffraction peak. This interpretation has further been discussed in the discussion section.

**Figure 6:**
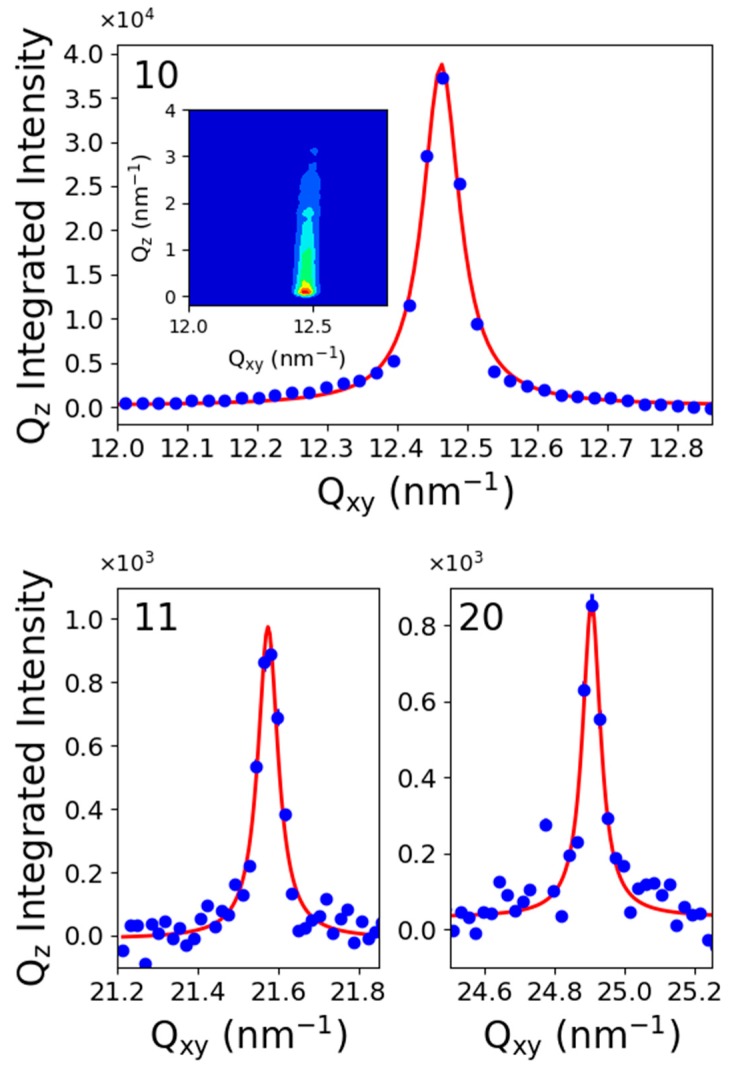
Diffraction spectra of C_11_F_23_CO_2_H at 18 °C and 15 mN/m. Top: (10) peak; Bottom (11) and (20) peaks.

Above 9 mN/m, one obtained a diffraction peak, which can be fitted by a single Lorentzian function. The position of the peak maximum ranged from 12.45 nm^−1^ to 12.57 nm^−1^, and its maximum intensity was located at Q_z_ = 0 nm^−1^, indicating vertical chains. Such single peak suggests a perfect hexagonal organization of the fluorinated chains with a lattice parameter ranging at a = 0.595 nm and an area of the unit cell (molecular area) of about 0.29 nm^2^. In such a hexagonal lattice, this peak was indexed as (10), and since the signal to noise ratio was over 50, we searched for higher-order ones. The (11) peak was expected at Q_11_ = √3 Q_10_ and the (20) peak at Q_20_ = 2 Q_10_. Figure 6-bottom presents the scattered intensity in the corresponding reciprocal space region. Indeed, one observed the higher index peaks at the expected position, confirming the perfect hexagonal organization of the fluorinated chains lattice over 9 mN/m. From the peak position, we computed the lattice parameters and the unit cell area when the surface pressure increased from 15 mN/m to 50 mN/m One obtained an area per molecule varying from 0.294 nm^2^ to 0.289 nm^2^ and finally microscopic lattice compressibility of about 0.5 mN/m, which is usually lower than the macroscopic one. We underlined that the second peak observed in the coexistence plateau followed perfectly this compressibility measured in this single phase (see Figure 4).

### 2.4. Molecular Dynamics Simulation Results

**Figure 7:**
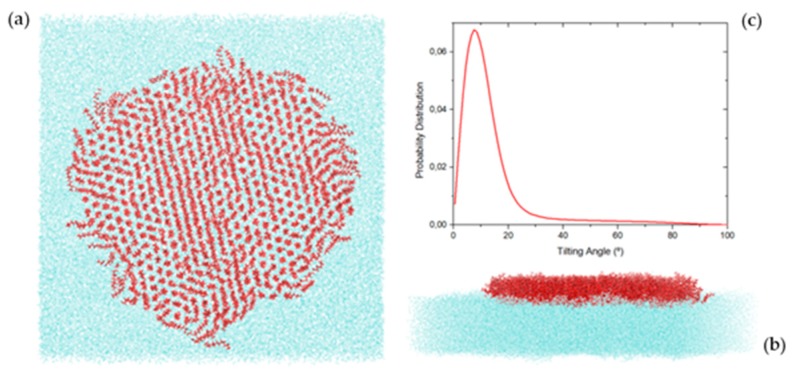
Snapshots of a simulation of 550 C_11_F_23_CO_2_H molecules monolayer at the surface of the water at 0.6 nm^2^/molecule and 298 K; (**a**) top-view; (**b**) side-view; (**c**) tilt angle distribution.

In order to obtain molecular-level insight into the studied system, atomistic molecular dynamics simulations were performed. As previously explained, the simulated system consisted of 550 PFDA molecules at the surface of the water, at an area per molecule of 0.6 nm^2^ (middle of the plateau) and 298 K. A typical snapshot of the simulation results is presented in Figure 7. As can be seen, the PFDA molecules clustered together in a single aggregate, although very few molecules could be observed dispersed as monomers throughout the surface of the water. A closer look of the cluster reveals the coexistence of two types of subdomains, one where the PFDA molecules were verticals and the other in which they were tilted. The tilting angle distribution for all the molecules is shown in Figure 7c (the axis of the molecule is defined by connecting the first H atom of the acid head group to the last F atom of the perfluorinated chain, leading to a non-zero angle with the normal to the surface when the molecule is vertical). Using this reference, the vertical PFDA molecules were centered at 10° and distributed on angles from 0° to 25°. The tilted molecules displayed a uniform distribution of angles between 25° and 90°. It is also very clear that the vertical molecules formed a hexagonal lattice, while tilted molecules tended to form lines parallel to each other. The formation of these lines indicates that the azimuthal angle was constant for these tilted molecules.

**Figure 8:**
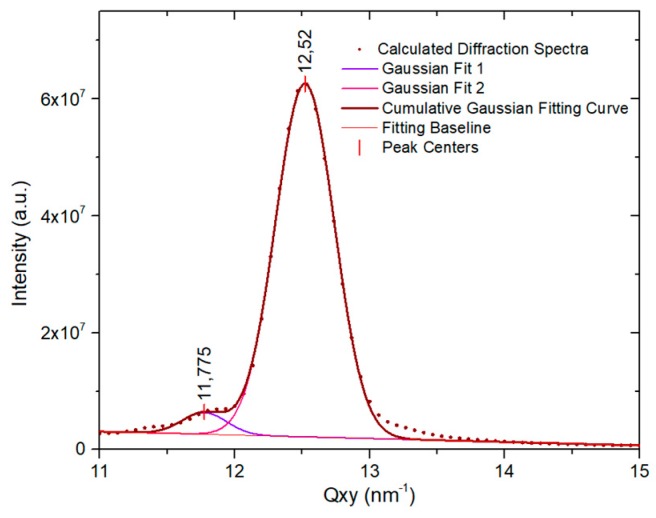
Calculated diffraction spectrum computed from molecular dynamics (MD) simulation of C11F23CO2H monolayer at 298.15 K of Figure 7.

The diffraction spectrum of the simulated system was also calculated from the simulation trajectories. The result is presented in Figure 8. In the calculated spectrum, an in-plane peak at Q_xy_ = 12.52 nm^−1^ was observed. It corresponded to the hexagonal structure and was in very good agreement with the experimental peak observed at 12.45 nm^−1^. A second peak observed at 11.78 nm^−1^ was due to the parallel line formed by the tilted molecules. The maximum was located at a lower value than the experimental peak, 12.25 nm^−1^, but the overall agreement between the calculated and experimental spectra could be considered quite good. It shows that the average distance between the lines of tilted molecules in the simulation was slightly larger than the experimentally measured. Also, the relative intensity of the two peaks was larger in the simulated spectra, indicating a different proportion of the domains formed by each type of molecules.

## 3. Discussion

First of all, it should be acknowledged that given the rigidity of perfluorinated chains, the organization of the less dense phase of these perfluorinated fatty acids monolayers could not be expected to be similar to that of liquid expanded (LE) phases observed for hydrogenated fatty acids monolayers [14]. This was first confirmed by the compressibility value, over 130 mN/m, much higher than that of about 50 m × N^−1^ observed for common LE phases of hydrogenated fatty acid [22]. The low-density fluid phase observed in the perfluorinated fatty acids isotherms could thus be a 2D Gas phase (G). At this point, it should be remembered that for monolayers of hydrogenated fatty acids, the onset of the G-LE transition is never observed, as it is expected to appear at extremely low densities (very large areas per molecule), and therefore very low 2D pressures. The state and organization of the low-density fluid phase in the case of perfluorinated acids are not obvious and should be analyzed in detail. Moreover, the transition to the liquid condensed phase seems more complex than previously described from lower resolution diffraction measurement [16,17], which means that the experimentally accessible temperatures correspond to higher reduced temperatures. As a consequence, it is thus plausible that the G-L plateau becomes experimentally detectable. We fitted the less dense phase region of the isotherm (before the plateau, Figure S1 Supplementary Information) with the 2D van der Waals equation $\left(\pi +\frac{a}{A^{2}}\right)\left(A-b\right)=R\times T$ (*π* is the surface pressure, *A* the area per molecule, *T* the temperature, and *R* the Boltzmann constant; *a* and *b* are adjustable parameters that model the molecular interactions, *a* quantifies attractive interactions, and *b* is the molecular co-surface, the minimum area occupied by a molecule). The obtained interaction parameter *a* = 1.21 × 10^9^ ± 4.8 × 10^7^ Pam^5^/mol^2^ lied in the range of typical van der Waals constants calculated for various organic molecules [23]. As for the co-surface value *b* = 0.38 ± 0.01 nm^2^, it lied between the molecular area expected for lying down molecules, about 1 nm^2^, and the one obtained by GIXD for vertical molecules, less than 0.30 nm^2^. This simple calculation indicates that this phase could be modeled as a 2D van der Waals fluid in which molecules occupy an average molecular area slightly larger than the one of vertical close-packed molecules. Considering the surface occupied by lying molecules and the co-surface obtained from the van der Waals adjustment of the isotherm, one obtained an average tilt angle of about 25°, not far from the ones deduced from those XRR measurements.

Two-dimensional assembly of rigid rods has been studied theoretically in the Gelbard-Ben-Shaul model (GBS). In this model, the rigid rods experience conformational energy, intermolecular interaction (attraction), and entropy [24,25], and the problem is treated using the Onsager–Zwanzig analysis [26,27]. They identified two transitions when increasing density, one gas to liquid (G ⇒ L) transition followed by standing up phase (Z). The vertical molecules, in the high-density phase Z, seem to be organized. The G ⇒ L transition was described using the van der Waals equation. Depending on the temperature, these two-phase transitions could be separated, G ⇒ L ⇒ Z, or merged into one single G ⇒ Z transition [25]. Given the rigidity and stiffness of the perfluorinated chains, these theoretical results obtained for rigid rods could be relevant for interpreting the experimental results of the real system. The observed plateau could thus represent the transition from the G or low-density L phase to the hexagonal LC phase. Since one of the diffraction peaks observed by GIXD on the plateau was attributed to the hexagonal structure of the LC phase, the other peak must result from the low-density fluid phase. This implies that this phase should at least be sufficiently organized to produce a diffraction peak. This assumption is compatible with the observed domains of parallel lines formed by tilted molecules obtained by the MD simulation. Such orientation of the molecule is in a first sight similar to the 3D isotropic-nematic phases transition in liquid crystal, as described by Onsager [26]. It should also be kept in mind that, contrarily to the “common” gaseous phase of hydrogenated amphiphiles, the surface density of this phase is of the same order as that of the LC phase. Given the aspect ratio of these rigid molecules, it seems reasonable that they tend to align in this fluid phase, even if they are not vertical. As mentioned above, the simulation results indicate that at the highest surface density of the fluid phase, tilted perfluorinated molecules tended to align spatially and azimuthally to form parallel lines. Such a structure, with an average orientational order of the molecules, could be ascribed as a 2D nematic phase since they are located in the plane. However, one does not expect translational order in a nematic phase. At 3D, the smectic phase structure is defined by parallel layers periodically stacked and containing disordered aligned molecules (with their center of mass located in the layer) and with a tilt angle that can vary (Smectic A if the director is perpendicular to the layers or Smectic C if it is tilted). The identified F phase is formed by parallel lines periodically stacked (in the plane) and formed by tilted molecules (for which the azimuth angle is along the line). However, since the tilted angle can vary into a line, the question arises whether such a phase could be associated to a nematic or a smectic phase. Such a question has been previously studied by considering the shape of the molecule [28], and the criterion is the variation of the order parameter with respect to the physical parameters (density, temperature, etc.) that is not yet performed for the PFDA monolayer. Then, considering the periodicity of the lines containing tilted molecules, we considered the F phase as a structure similar to a “2D smectic C”, each line in the 2D space acting as a plane of molecules in the 3D space.

**Figure 9:**
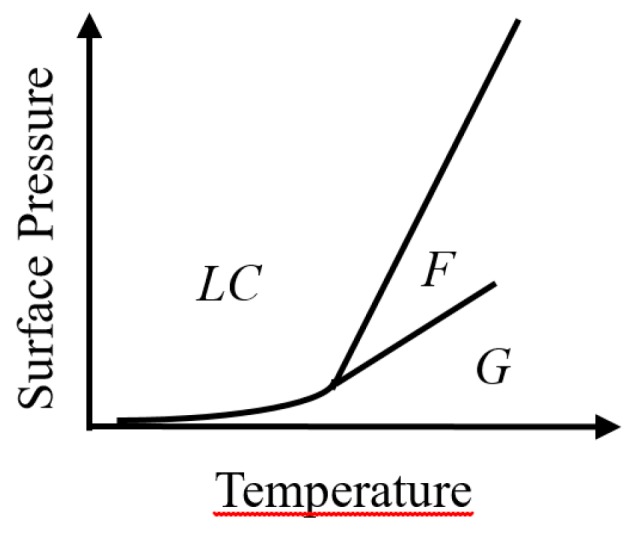
Qualitative phase diagram of the monolayer formed by rigid perfluorinated amphiphilic molecules: G, 2D gas; F, 2D smectic C monolayer phase; LC, a hexagonal lattice of vertical chains.

Of course, at very low surface density, the existence of a 2D Gas-phase can be expected. The question raised is how the transition between the two phases (fluid and LC) evolves at a higher temperature since, above a critical temperature, one expects a second-order transition. However, a second-order transition between two distinct phases is not possible with symmetry breaking [29], which is not the case for the G-LC transition. This new intermediate 2D smectic C phase then allows the possibility of the two successive second-order phase transition [30]. Thus, three phases should be considered in the phase diagram of monolayers formed by perfluorinated molecules (Figure 9): The G (gas) phase not measured at the densities explored in this work, the F phase (similar to a 2D smectic C), and the LC (Liquid condensed). The expected two successive phases transitions, G ==> F ==> LC, would be difficult to check experimentally.

## 4. Materials and Methods

### 4.1. Molecules and Sample Preparation

Perfluorododecanoic acid (Aldrich, purity ≈ 95%) was dissolved in hexane (Fluka-HPLC grade-Honeywell Specialty Chemicals Seelze GmbH, Seelze, Germany) / ethanol (Fisher-HPLC grade-Fisher Scientific SAS, Illkirch, France) (9/1 *v/v*). Each solution was spread at the surface of an aqueous subphase (Millipore water, Merck Millipore, Darmstadt, Germany) in a Langmuir trough Surface. The pH was adjusted to 1.5–2 by adding HCl to the water. The pressure was measured by the Wilhelmy plate method [14] using a Riegler and Kirstein Gmbh (Berlin, Germany) pressure sensor. The temperature of the subphase was controlled by a water circulation bath and varied from 12 °C to 24 °C. The film was continuously compressed at a constant rate of 0.02 nm^2^/molecule/min after allowing 10 to 15 min for the spreading solvent to evaporate.

### 4.2. X-ray Reflectivity (XRR)

The XRR experiments were performed at the I22 beamline at the Diamond synchrotron source. The energy of the incident X-ray was 12.5 keV (λ = 1 nm), and the beam size was 0.1 × 2 mm^2^ (V × H) at the sample position. The scattered intensity was collected with a Pilatus 100k pixel detector (Dectris, Switzerland) in the specular geometry. The images were horizontally integrated to obtain the reflected peak. Each reflection was fitted by a Gaussian shape function and a sloping background. The peak intensity was then used to build the reflectivity curve with respect to the incidence angle. A Nima Langmuir through was installed on anti-vibration active system to reduce the liquid surface fluctuations and enclosed in a gas-tight box flushed by a continuous helium flux to reduce the diffuse air scattering. Detail of the experimental setup can be found in reference [31].

### 4.3. Grazing Incidence X-ray Diffraction (GIXD)

GIXD experiments were performed on the SIRIUS beamline at the SOLEIL synchrotron source. The details and the optics of the facility are described in reference [19]. The energy of the incident X-ray was 8 keV (λ = 0.155 nm), and the beam size was 0.1 × 2 mm^2^ (V × H) at the sample position. The surface was illuminated at an incident angle of 2 mrad, below the critical angle of the air-water interface (2.8 mrad at 8 keV). Thus, the incident wave was almost totally reflected, while the refracted wave became evanescent, leading to a probed thickness of about 5 nm beneath the interface. The scattered intensity was collected with very low noise, using a 1D gas detector fixed on the 2-axis detector arm of the beamline’s diffractometer. A Soller slit collimator was positioned in front of the detector, leading to an in-plane wave vector resolution of 0.03 nm^−1^ in the Q_xy_ range recorded.

### 4.4. Simulation Detail

Molecular Dynamics simulations were done for perfluorododecanoic acid monolayers at the surface of the water, using GROMACS 5.0.7 [32,33]. PFDA molecules were modeled using the general Optimized Potentials for Liquid Simulations (OPLS) framework [34] for the polar COOH group, and the perfluoroalkyl segment was modeled according to the parameters described in ref. [35]. Following previous work for the n-alkane family for which a specific version for long n-alkanes was developed, L-OPLS, in the case of the perfluoroalkyl segment, the partial charges of the C and F atoms and the L-J dispersive energy parameter, ε, were slightly modified to account for the long 12 carbons chain. The value of the partial charge of fluorine was taken within the range of those obtained for PFDA by quantum mechanical calculations with Orca 4.0.1 software [36] at the PBE0/6-311++g(d,p) level of theory, using the ChelpG formalism. The adopted value, Q_F_ = −0.15, was 25% more negative than the original OPLS value (QF = −0.12). As usual, the partial charge of carbons was assumed to compensate the sum of the fluorine charges directly bonded to each carbon. The adopted ε value of fluorine (ε_FF_ = 0.208 kJ × mol^−1^) was 6% lower than the original OPLS value (ε_FF_ = 0.222 kJ × mol^−1^) and reproduced the experimental vaporization enthalpy of perfluorononane (45270 J/mol), the longest perfluoroalkane for which accurate data is available. An 8% change in the Lennard–Jones energy parameter of fluorine did not affect the overall picture presented by the simulation results. The SPC/E model was used for the water molecules [37]. Geometric mean rules were used to compute the potential parameters for the van der Waals interactions between atoms of a different kind.

**Figure 10:**
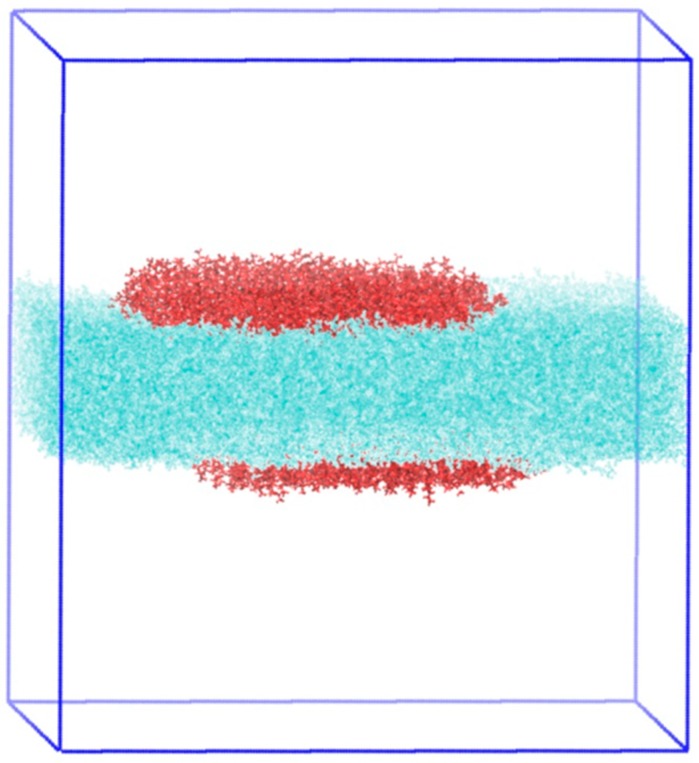
Typical simulation box for perfluorododecanoic acid (PFDA) monolayers at the vacuum-water interface.

All simulation boxes were designed with a layer of 40,000 water molecules with a geometry of 18.2 × 18.2 × 20 nm^3^ in order to present a layer thickness of about 3.5 nm and two available liquid-vapor interfaces to localize the amphiphilic molecules. The simulation boxes were equilibrated in the NVT ensemble during 1 ns. Using the Packmol software package [38], two sets of 550 PFDA molecules were closely and vertically deposited on the water surface with the carboxylic groups facing the water interface; thus, the simulations were run at an area/molecule of PFDA of 0.6 nm^2^/molecule. The equilibration run was done during 10 ns in NVT ensemble, followed by a 5 ns NVT production run. A typical simulation box is shown in Figure 10.

All the simulations were done using a time-step of 2 fs and cut-off values of 1.4 nm for van der Waals and electrostatic interactions. For distances beyond the cut-off, long-range electrostatic and Lennard–Jones interactions were calculated using the Particle Mesh Ewald (PME) method. The simulations were done at 298.15 K, using V-Rescale thermostats in sequence with coupling constants of 0.5 ps [39].

## 5. Conclusions

New experiments on Langmuir monolayers of C_11_F_23_CO_2_H perfluorinated fatty acid enabled a more accurate determination of the Langmuir isotherm and characterization of the observed phases and their transitions. X-ray reflectivity and grazing incidence X-ray diffraction measurements performed on the transition plateau to the LC phase with a higher resolution instrument provided evidence for the existence of “2D smectic C phase” that was not previously detected. This new phase was believed to result from the relatively weak intermolecular interactions between the perfluorinated chains associated with their rigidity and large aspect ratio. Indeed, weak intermolecular interactions induced a relatively low critical temperature for the transition to the LC phase, increasing the reduced temperature at which the experiments are performed. This resulted in a small width of the coexistence plateau and thus a relatively high density of the coexisting fluid phase. At such high density, the average area per molecule was necessarily lower than occupied by molecules lying parallel to the surface. This led to a tilting of the chains, which were associated with their stiffness and large aspect ratio, resulting in an orientation and packing process, leading to the appearance of parallel lines, forming the 2D smectic C organization. Atomistic molecular dynamics simulations provided a molecular-level interpretation of the results and visualization of the studied system. Moreover, the diffraction spectrum calculated from the simulation results compared favorably with the experimental pattern, fully validating the simulations and the proposed interpretations. In conclusion, three phases G, F, and LC should be considered in the phase diagram of monolayers formed by rigid chain fluorinated molecules.

## Figures and Tables

**Table 1:** Macroscopic compressibility of the two phases, Fluid Phase and Liquid Condensed (LC) phase calculated from the surface pressure-area per molecule (π-A) isotherms.

Temperature (°C)	Fluid Phase (m/N)	LC Phase (m/N)
24	130–300	8 ± 0.2
20	160–300	8.5 ± 0.2
18	190–300	7.3 ± 0.2
12	200–300	13 ± 0.2

## Supplementary Materials

The following are available online at https://www.mdpi.com/1420-3049/24/19/3590/s1, Figure S1: Adjustment of the fluid phase region of the isotherm by the Van der Waals equation.

## References

[1] Karsa D.R. (1994). Fluorinated Surfactants: Synthesis Properties Applications, by Erik Kissa.

[2] Cappelletti G., Ardizzone S., Meroni D., Soliveri G., Ceotto M., Biaggi C., Benagia M., Raimond L. (2013). Wettability of bare and fluorinated silanes: A combined approach based on surface free energy evaluations and dipole moment calculations. J. Colloid Interface Sci..

[3] Szwajca A., Koroniak H. (2016). Fluorinated SAMs on Si(001) surface; Surface electronic properties and structural aspects. Phosphorus Sulfur Silicon Relat. Elem..

[4] Riess J.G. (2001). Oxygen carriers (blood substitutes)—Raison d’être, chemistry and some physiology. Chem. Rev..

[5] Riess J.G. (2009). Highly fluorinated amphiphilic molecules and self-assemblies with biomedical potential. Curr. Opin. Colloid Interface Sci..

[6] Gerber F., Krafft M.P., Vandamme T.F., Goldmann M., Fontaine P. (2007). Potential use of fluorocarbons in lung surfactant therapy. Artif. Cells Blood Substit. Biotechnol..

[7] Schnutt E.G., Klein D.H., Mattrey R.M., Riess J.G. (2003). Injectable microbubbles as contrast agents for diagnostic ultrasound imaging: The key role of perfluoro chemicals. Angew. Chem..

[8] Steven P., Scherer D., Krösser S., Beckert M., Cursiefent C., Kaercher T. (2015). Semifluorinated alkane eye drops for treatment of dry eye disease: A prospective, multicenter, non-interventional study. J. Ocul. Pharmacol. Ther..

[9] Kirsch P. (2004). Modern Fluoroorganic Chemistry.

[10] Bunn C.W., Lowells E.R. (1954). Structure of molecules and crystals of fluorocarbons. Nature.

[11] Jang S.S., Blanco M., Goddard W.A., Caldwell G., Ross R.B. (2003). The Source of Helicity in Perfluorinated N-Alkanes. Macromolecules.

[12] Costa J.C., Lima C.F., Mendes A., Santos L.M. (2016). Fluorination effect on the thermodynamic properties of long-chain hydrocarbons and alcohols. J. Chem. Thermodyn..

[13] Silva G.M., Morgado P., Haley J.D., Montoya V.M.T., McCabe C., Martins L.F., Filipe E.J. (2016). Vapor pressure and liquid density of fluorinated alcohols: Experimental, simulation and GC-SAFT-VR predictions. Fluid Phase Equilibria.

[14] Gaines G.L. (1966). Insoluble Monolayers at Liquid-Gas Interfaces.

[15] Kaganer V.M., Möhwald H., Dutta P. (1999). Structure and phase transitions in Langmuir monolayers. Rev. Mod. Phys..

[16] Acero A.A., Li M., Lin B., Rice S.A., Goldmann M., Ben Azouz I., Goudot A., Rondelez F. (1993). Molecular packing in water supported monolayers of F(CF_2_)_11_COOH and F(CF_2_)_10_CH_2_COOH. J. Chem. Phys..

[17] Goldmann M., Nassoy P., Rondelez F., Renault A., Shin S., Rice S.A. (1994). In-plane X-ray diffraction from monolayers of perfluorinated fatty acids: Evidence for azimuthal ordering in the condensed phase. J. Phys. II.

[18] Schmidt M.E., Shin S., Rice S. (1996). Molecular dynamics studies of Langmuir monolayers of F(CF_2_)_11_COOH. J. Chem. Phys..

[19] Fontaine P., Ciatto G., Aubert N., Goldmann M. (2014). Soft Interfaces and Resonant Investigation on Undulator Source: A Surface X-ray Scattering Beamline to Study Organic Molecular Films at the SOLEIL Synchrotron. Sci. Adv. Mater..

[20] Braslau A., Deutsch M., Pershan P.S., Weiss A.H. (1985). Surface Roughness of Water Measured by X-ray Reflectivity. Phys. Rev. Lett..

[21] Braslau A., Pershan P.S., Swislow G., Ocko B.M., Als Nielsen J. (1988). Capillary waves on the surface of simple liquid measured by X-ray reflectivity. Phys. Rev. A.

[22] Yu Z.W., Jin J., Cao Y. (2002). Characterization of the liquid-expanded to liquid-condensed phase transition of monolayers by means of compressibility. Langmuir.

[23] Atkins P.W., De Paula J., Keeler J. (2018). Physical Chemistry.

[24] Chen Z.-Y., Talbot J., Gelbart W., Ben-Shaul A. (1998). Phase Transitions in Systems of Grafted Rods. Phys. Rev. Lett..

[25] Kramer D., Ben-Shaul A., Chen Z.-Y., Gelbart W.M. (1992). Monte-Carlo and Mean-Field Studies of Successive Phase Transitions in Rod Monolayers. J. Chem. Phys..

[26] Onsager L. (1949). The Effects of Shape on the Interaction of Colloidal Particles. Ann. N. Y. Acad. Sci..

[27] Zwanzig R. (1963). First-Order Transition in a Gas of Long Thin rods. J. Chem. Phys..

[28] Szabolcs V., Gurin P., Armas-Pérez C.J., Quintana H.J. (2009). Nematic and smectic ordering in a system of two dimensional hard zigzag particles. J. Chem. Phys..

[29] Landau L.D., Lifschitz E.M. (1959). Statistical Physics.

[30] Lajzerowicz J., Sivadière J. (1974). Spin-1 lattice gas model I. Condensation and solidification of a simple fluid. Phys. Rev. A.

[31] Arnold T., Nicklin C., Rawle J., Sutter J., Bates T., Nutter B., McIntyre G., Burt M. (2012). Implementation of a beam deflection system for studies of liquid interfaces on beamline I07 at Diamond. J. Synchrotron Rad..

[32] Van Der Spoel D., Lindahl E., Hess B., Groenhof G., Mark A.E., Berendsen H.J.C. (2005). GROMACS: Fast, Flexible, and Free. J. Comput. Chem..

[33] Pronk S., Páll S., Schulz R., Larsson P., Bjelkmar P., Apostolov R., Shirts M.R., Smith J.C., Kasson P.M., van der Spoel D. (2013). GROMACS 4.5: A high-throughput and highly parallel open source molecular simulation toolkit. Bioinformatics.

[34] Jorgensen W.L., Maxwell D.S., Tirado-Rives J. (1996). Development and Testing of the OPLS All-Atom Force Field on Conformational Energetics and Properties of Organic Liquids. J. Am. Chem. Soc..

[35] Watkins E.K., Jorgensen W.L. (2001). Perfluoroalkanes: Conformational Analysis and Liquid-State Properties from Ab Initio and Monte Carlo Calculations. J. Phys. Chem. A.

[36] Neese F. (2012). The ORCA program system. Comput. Mol. Sci..

[37] Berendsen H.J.C., Grigera J.R., Straatsma T.P. (1987). The missing term in effective pair potentials. J. Phys. Chem..

[38] Martínez L., Andrade R., Birgin E.G., Martínez J.M. (2009). Packmol: A Package for Building Initial Configurations for Molecular Dynamics Simulations. J. Comput. Chem..

[39] Bussi G., Donadio D., Parrinello M. (2007). Canonical Sampling through Velocity-Rescaling. J. Chem. Phys..

