# Supplementary figures and images for: Structure of Langmuir Monolayers of Perfluorinated Fatty Acids: Evidence of a New 2D Smectic C Phase
Source: Molecules. 2019 Oct 5;24(19):3590. doi: 10.3390/molecules24193590 (PMC6804139; doi:10.3390/molecules24193590)

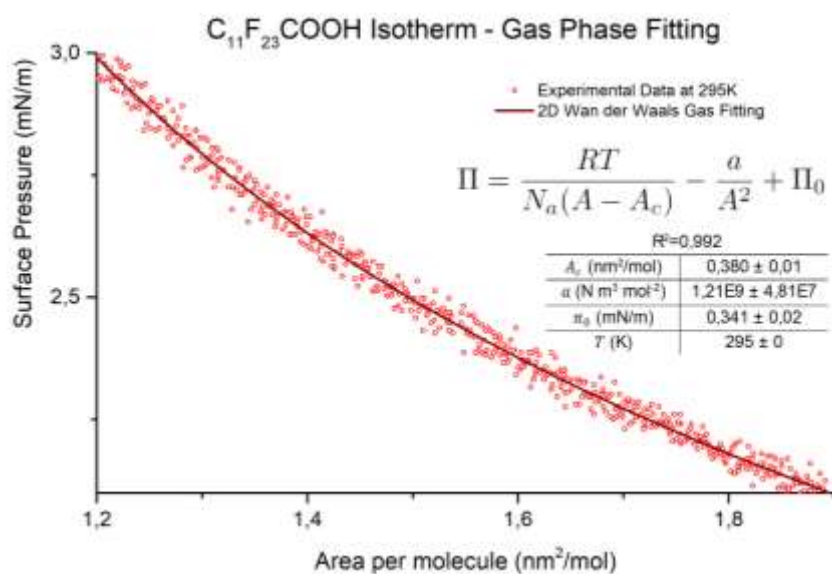

Figure S1: Adjustment of the fluid phase region of the isotherm by the Van der Waals equation.

Supplement: Supplementary file 1 [file molecules-24-03590-s001.pdf]
